# *Brucella* species-induced brucellosis: Antimicrobial effects, potential resistance and toxicity of silver and gold nanosized particles

**DOI:** 10.1371/journal.pone.0269963

**Published:** 2022-07-14

**Authors:** Ayman Elbehiry, Musaad Aldubaib, Osamah Al Rugaie, Eman Marzouk, Ihab Moussa, Mohamed El-Husseiny, Mai Ibrahem, Adil Abalkhail, Mohammed Rawway

**Affiliations:** 1 Department of Public Health, College of Public Health and Health Informatics, Qassim University, Al-Bukairiyah, Saudi Arabia; 2 Department of Bacteriology, Mycology and Immunology, Faculty of Veterinary Medicine, University of Sadat City, Sadat City, Egypt; 3 College of Agriculture and Veterinary Medicine, Qassim University, Qassim, Saudi Arabia; 4 Department of Basic Medical Sciences, College of Medicine and Medical Sciences, Qassim University, Unaizah, Qassim, Saudi Arabia; 5 Department of Botany and Microbiology, College of Science, King Saud University, Riyadh, Saudi Arabia; 6 Department of Microbiology, Faculty of Veterinary Medicine, Cairo University, Giza, Egypt; 7 Animal Health Research Institute—Agriculture Research Center, Giza, Egypt; 8 Department of Public Health, College of Applied Medical Science, King Khalid University, Abha, Saudi Arabia; 9 Department of Fish Diseases and Management, Faculty of Veterinary Medicine, Cairo University, Cairo, Egypt; 10 Biology Department, College of Science, Jouf University, Sakaka, Saudi Arabia; 11 Botany and Microbiology Department, Faculty of Science, AL-Azhar University, Assiut, Egypt; King Abdulaziz University, SAUDI ARABIA

## Abstract

Brucellosis is an endemic zoonotic disease caused by *Brucella* species, which are intramacrophage pathogens that make treating this disease challenging. The negative effects of the treatment regime have prompted the development of new antimicrobials against brucellosis. A new treatment modality for antibiotic-resistant microorganisms is the use of nanoparticles (NPs). In this study, we examined the antibacterial activities of silver and gold NPs (SNPs and GNPs, respectively), the resistance developed by *Brucella melitensis* (*B*. *melitensis*) and *Brucella abortus* (*B*. *abortus*) strains and the toxicity of both of these NPs in experimental rats. To test the bactericidal effects of the SNPs and GNPs, we used 22 multidrug-resistant *Brucella* isolates (10 *B*. *melitensis* and 12 *B*. *abortus*). The minimal inhibitory concentrations (MICs) of both types of NPs were determined utilizing the microdilution technique. To test the stability of resistance, 7 *B*. *melitensis* and 6 *B*. *abortus* isolates were passaged ten times in culture with subinhibitory concentrations of NPs and another ten times without NPs. Histopathological analysis was completed after rats were given 0.25, 0.5, 1, and 2 mg/kg NPs orally for 28 consecutive days. The MIC values (μg/ml) of the 10-nm SNPs and 20-nm GNPs against *B*. *melitensis* were 22.43 ± 2.32 and 13.56 ± 1.22, while these values were 18.77 ± 1.33 and 12.45 ± 1.59 for *B*. *abortus*, respectively. After extensive *in vitro* exposure, most strains showed no resistance to the 10-nm SNPs or 20-nm GNPs. The NPs and antibiotics did not cross-react in any of the evolved *Brucella* strains. SNPs and GNPs at doses below 2 mg/kg were not harmful to rat tissue according to organ histopathological examinations. However, a greater dose of NPs (2 mg/kg) harmed all of the tissues studied. The bactericidal properties of NPs are demonstrated in this work. *Brucella* strains develop similar resistance to SNPs and GNPs, and at low dosages, neither SNPs nor GNPs were hazardous to rats.

## Introduction

Infectious diseases continue to be one of the most common causes of death in humans and animals worldwide [1]. The World Health Organization (WHO) and the Centers for Disease Control and Prevention (CDC) have highlighted grave concerns regarding the continuous rise in the numbers of multidrug-resistant microorganisms [2, 3]. As a result, among the most important challenges in health care is drug resistance [4]. The absence of novel antimicrobial compounds is related to the emergence of bacterial resistance [5]. This has prompted efforts around the world to create new and more efficient antimicrobial compounds, along with new administration tactics [1].

Brucellosis is a resurgent bacterial endemic infectious disease that affects domestic and wild animals as well as humans. Mammalian brucellosis is expected to affect approximately one million people each year, with 40% of cases progressing to chronic illness [6, 7]. A mixture of antibiotics is the recommended treatment for human brucellosis induced by clinical strains of *Brucella melitensis* (*B*. *melitensis*) and *Brucella abortus* (*B*. *abortus*) [8–10]. Nevertheless, there is a significant probability of the recurrence of illness and therapeutic failure due to increasing resistance; thus, the fundamental concern for brucellosis management is that the pathogenesis of the condition is important [7]. As a result, a new approach to treat brucellosis is greatly needed.

Because *B*. *melitensis* is a highly pathogenic species of *Brucella* that infects humans, brucellosis management is a difficult task [11]. The intracellular location of this bacterium makes its removal extremely difficult. Furthermore, Gamazo et al. [12] discovered that numerous antibiotics lose their efficacy to effectively target *Brucella*-infected cells due to their inability to persist for long enough to produce a therapeutic effect. As a result, current brucellosis treatment for both animals and humans necessitates the use of a long-acting antimicrobial drug. For the treatment of human brucellosis, a previous study [13] recommended a combination of two types of antibiotics, doxycycline and rifampicin, for at least six weeks. Another study carried out by Al Barraq et al. [14] suggested administration of aminoglycosides and streptomycin for two to three weeks as an appropriate antibiotic combination for brucellosis treatment. Despite the realistic efficacy of antibiotic therapy, it is frequently ineffective in eradicating the disease, with relapse rates of approximately 5–10% [15]. To address the problem of recurring infection caused by antibiotic-resistant *Brucella* species, particularly *B*. *melitensis*, a new medication to eliminate intracellular pathogens has been approved [16]. As a result, utilizing nanoparticles (NPs) to treat *Brucella*-infected cells may lead to prolonged intracellular deposition of this antibiotic agent, which will solve the problem of relapse [17].

Technological breakthroughs in nanotechnology have opened up new possibilities for developing novel compounds depending on the type, shape, and size of the NPs with variable antibacterial capabilities [7, 18]. The use of NPs could be a feasible tactic because they can both fight microorganisms and serve as transporters for antibiotics and natural antibacterial substances [19]. The large surface-to-volume ratio of bactericidal NPs leads to their potential targets, which further minimizes the risk of drug resistance [20–22].

The use of NPs has recently been utilized as another approach to treat a variety of antimicrobial-resistant pathogens, and they could potential fix the problem of multidrug resistance [19, 23]; in particular, silver nanoparticles (SNPs) have received much interest [24–26] because of their low cytotoxic effects [27, 28], and silver has historically been used as an effective antibacterial against a variety of microorganisms [29].

The antimicrobial activities of SNPs were recently investigated [30]. According to different articles, SNPs have a larger surface area than silver ions and are therefore more potent antimicrobial compounds. SNPs are among the most frequently examined NPs in nanotechnology because of their particular pharmacological, morphological, and biological characteristics [31, 32]. SNPs have been synthesized using a variety of approaches, comprising physical, chemical, and biological methods. Nevertheless, a rising demand for sustainable alternatives and the use of biodegradable ingredients has piqued interest in the bioremediation process of synthesizing NPs [33, 34]. Numerous species of bacteria could truly show tolerance to silver and SNP therapies even though they have been represented as antibacterial agents for several resistant pathogens according to published findings [35–38], whereas other research has come to different conclusions.

The antibacterial effect of gold nanoparticles (GNPs) was recently revealed to be augmented by their relatively greater surface area/volume [22, 39, 40]; thus, GNPs have stronger antibacterial activity than gold by itself [39, 41, 42]. Several studies have shown that GNPs have antimicrobial properties against human pathogens; however, the minimum inhibitory concentration (MIC) values for 7-nm and 16-nm GNPs against *Escherichia coli* and *Staphylococcus aureus* were not statistically significant [43, 44].

In general, NPs with characteristics such as a high specific surface area and a capacity to deliver a large number of antimicrobial drugs or other compounds have been found to be suitable antibacterial agents [45–48]. These characteristics, whether visible or not, may enhance the antibacterial activity. Nanoparticle size is a property that endows a material with antibacterial activity. Aside from their inherent antibacterial properties, the extremely large surface area of NPs plays an essential role in microbial adhesion and quick invasion into cells as noted by a variety of studies [48–50].

Several mechanisms of pathogen resistance to antibiotics are becoming less effective since NPs may destroy them by adhering directly to their cellular components without penetration. This raises the likelihood that NPs will cause less bacterial resistance than antimicrobial drugs [19]. In general, cellular oxidative development [51], metal ion liberation [52], and nonoxidative processes [53] are the antibacterial mode of actions of NPs. Notably, these mechanisms could all happen at approximately the same moment. Repeated exposure to sublethal dosages of biocontrol agents, on the other hand, can result in the development of multidrug efflux and bacterial resistance [54].

Few investigations on bacteria have been undertaken to determine whether their tolerance to sublethal dosages of NPs ultimately leads to antibiotic resistance [22, 55, 56]. While certain reports suggest that NPs and antibiotics can establish cross-resistance, others claim that no such correlation exists [22, 57, 58]. The toxic influence of NPs is a key concern in the field of nanomaterials since they are widely diffused in air, health care settings, and perhaps even foodstuffs [59].

Despite the fact that the potential impacts of NPs have attracted attention, few experiments on the side-effects have been carried out [59]. Only few studies have analyzed the hazardous properties of NPs on the digestive system, on which they reported signs of toxicity [60–62]. Furthermore, clinical studies have identified that a variety of different types of medical equipment emit silver ions into circulation, concentrating them in various parts of the body (e.g., intestines, liver, pancreas, brain, lung, kidney), resulting in serious damage and mortality [63]. SNPs and GNPs are thought to cause cytotoxicity, although the mechanism is unknown [22]. Nevertheless, another investigation found that SNPs accumulated in the splenic, hepatic, and renal tissues of rats, implying that SNPs were transported to and accumulated in particular target tissues where they may release Ag+ [64].

Considering the abovementioned earlier results, we feel that investigating the antibacterial and undesirable consequences of NPs is a critical and important issue. Therefore, our goal is to analyse the *in vitro* bactericidal and cytotoxic effects of SNPs and GNPs against multidrug-resistant *Brucella* species, as well as the *in vivo* toxicity of both nanosized particles following four weeks of oral administration to experimental rats.

## Materials and methods

### Ethics statement

In terms of sample collection, there were no human or animal participants, so neither ethical approval nor written consent was required. All of the clinical strains used in this study came from regular medical testing or strain collections, and only bacterial cultures obtained from these sources were used. In the study of the toxic effects of SNPs and GNPs on experimental rats, all procedures were conducted according to the National Institute of Health Guide for the Care and Use of Laboratory Animals and the Ethics Rules of the Laboratory Animal Care Center at Qassim University, KSA. Ethical approval no. 21-12-30 was obtained from the Ethics Committee of the Deanship of Scientific Research at Qassim University, Saudi Arabia. In all of the experimental procedures, rats were anaesthetized using ketamine-xylazine to minimize pain and suffering.

### Bacterial isolates

In this investigation, 25 *Brucella* species isolates (11 *B*. *melitensis* and 14 *B*. *abortus*) were recovered from 364 animal samples with a high occurrence of brucellosis and 70 human blood samples obtained from individuals suffering from hyperthermia who had close contact with suspect animals in the Al-Qassim province of Saudi Arabia.

### Identification of *Brucella* species

Selective *Brucella* agar (SBA)-containing antibiotics were utilized as the selective media for *Brucella* species culture. Testing was performed using probable strains of short coccobacilli with very small colonies that were both catalase- and oxidase-positive to determine the formation of urease and H_2_S. Agar plates (Sigma–Aldrich, Saint Louis, USA) were used to identify the isolates. A Vitek 2 instrument and GN cards (bioMe’rieux, France) were used as directed by the supplier. The rapid slide agglutination antigen and a complement-enzyme linked immunosorbent assay (cELISA) were used to identify the *Brucella* species obtained. As a confirmatory method of detection, a MALDI Biotyper (MBT) device was employed, as well as SYBR green real-time PCR analysis, which was verified by microfluidic electrophoresis investigations.

### NPs utilized in our investigation

**Fig 1:**
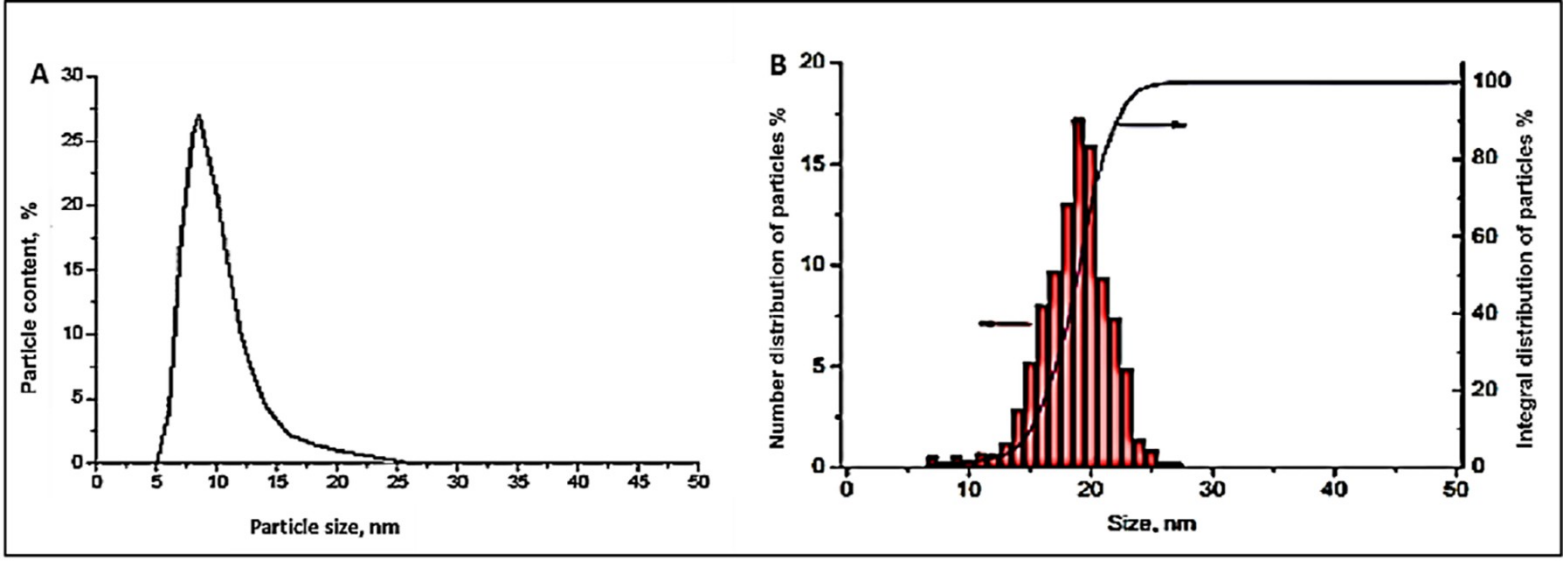
Size distribution of 10-nm SNPs (A) and 20-nm GNPs (B).

PlasmaChem GmbH (Berlin, Germany) provided us with two aqueous colloidal solutions of 10-nm SNPs and 20-nm GNPs to utilize in our research (Table 1 and Fig 1). Both NPs were synthesized using a synthetic decomposition method. In summary, SNPs were made utilizing silver nitrate as the silver precursor, the reduction reagent sodium borohydride (in solution), and polyvinylpyrrolidone (PVP) as the particle stabilizing mediator to avoid NP build-up. To minimize the level of impurities in the NPs, silver nitrate was prepared in distilled water, and PVP and sodium citrate tribasic hydrate were subsequently supplied. The mixture was then agitated for 30 minutes with the sodium borohydride solution. the production of the SNPs was detected by the change in the colour of the solution from transparent to brown. In addition, 10 mM tetrachloroauric acid (HAuCl4) was reduced with sodium citrate to produce GNPs. An aqueous gold (III) chloride trihydrate (HAuCl_4_.3H_2_O) solution was heated under pressure with agitation. Following the addition of 10 ml of trisodium citrate (1%) to the solution, the colour changed from yellow to red, indicating the creation of hemispheric GNPs. The solution was allowed to cool at 25°C after refluxing for 20 minutes. The product was filtered through a 0.45 μm acetate filter and kept at 4°C. Transmission electron microscopy (TEM) was used to investigate the structures of the produced NPs. The average diameters of the NPs were determined with a Malvern Zetasizer Nano ZS® instrument and by dynamic light scattering (Sysmex, Netherlands).

**Table 1:** Physicochemical properties of the SNPs and GNPs.

Property	Average value for SNPs	Average value for GNPs
Concentration	0.1 mg/ml	0.05 mg/ml
Average particle size	10 nm	20 ± 3 nm
pH	6–8	approximately 8
Stabilizer on the particle surface	Citrate	Citrate
Appearance	Yellow to green transparent solution	Red transparent solution

### Bactericidal effects of the SNPs and GNPs against multidrug-resistant *Brucella* species

Using the European Committee on Antimicrobial Susceptibility Testing (EUCAST) guidelines, we determined the minimum inhibitory concentrations (MICs) of SNPs and GNPs against 10 *B*. *melitensis* and 12 *B*. *abortus* strains by broth microdilution assay in 96-well microplates. Stock solutions of SNPs and GNPs at concentrations of 400 μg/4 ml (equivalent to 0.1 mg/ml) and 400 μg/8 ml (equivalent to 0.05 mg/ml), respectively, were prepared. A twofold dilution series (200, 100, 50, 25, 12.5, 6.25, 3.125, 1.56, 0.78 and 0.39 μg/ml) for each NP was prepared from a stock solution. Briefly, the 96-well microtiter plate was filled with 100 μl of sterile Mueller Hinton Broth (MHB) supplemented with 1% sheep haemoglobin (Hb) per well. The two control rows were the sterile control (200 μl of sterile MHB served as the sterility indicator) and culture control (100 μl of sterile MHB and 100 μl of subculture broth), which were used to assess the broth’s ability to maintain bacterial growth. The MIC of each type of NP was determined by placing 100 μl of the NP working solution at a given concentration into the top row of a 96-well microtiter plate and serially diluting by twofold across the NP test range. Then, 100 μl of culture broth adjusted to 0.5 McFarland standard was added to each row of wells except the sterile control row. Both types of NPs were tested in triplicate to ensure accuracy. After confirmation of the reliability, each plate was covered with sterile adhesive tape and incubated at 37°C for 24 hours, and the results were reported when the culture control row achieved sufficient turbidity and the sterile control row did not show turbidity or growth. MICs were determined by comparing each well’s growth to that of the control, and the lowest concentration of NP that completely inhibited growth was determined to be the MIC. A UV spectrophotometer (OD_600_) was used to measure the bactericidal effects of the NPs on bacterial growth according to CLSI (Clinical and Laboratory Standards Institute) and EUCAST guidelines. Based on the following equation, we calculated the bacterial growth inhibition percentage; % of growth inhibition = OD(I)-OD(II)/OD(I) x 100, where (I) is optical density (OD) of *Brucella* strain at 600 nm and (II) is OD of *Brucella* strain incubated with NPs determined at 600 nm.

To assess the minimum bacterial concentration (MBC), 50 μl aliquots from nonturbid wells were placed on selective Brucella agar (SBA) plates that had not been treated with NPs and cultured for 24 hours at 37°C. Before and after incubation, all plates were tested for the presence or lack of bacterial growth. The absence of bacterial growth on the plates indicated that the NP dose was lethal. The number of viable cells was used to determine the number of organisms that survived. The MBC was determined as the lowest NP dose that stopped the development of 99% of the *Brucella* species.

### Assessment of the development of resistance in *Brucella* species by treatment with 10-nm SNPs and 20-nm GNPs

This study included 22 *Brucella* strains (10 *B*. *melitensis* and 12 *B*. *abortus*) that had previously been treated with SNPs and GNPs. To promote resistance, isolates with lower MIC values were selected. *Brucella* strains were repeatedly passaged on nutrient broth with sublethal dosages of NPs below the MIC at quantities in which the isolates continuously grew in an attempt to encourage resistance. As a result, the concentrations of 10-nm SNPs and 20-nm GNPs utilized throughout the present investigation were 12.5 μg/ml and 6.25 μg/ml, respectively. Before and after 10 passages, the MICs in each treatment group were analysed. The strains were subcultured 10 times in the specified media with sublethal dosages of both SNPs and GNPs for 72 h periods under totally sterile conditions.

### NPs and antibiotic cross-resistance evaluation

To test the cross-resistance of *Brucella* strains resistant to NPs against various antibiotics, broth microdilution was used according to the M7-A8 recommendations of the Clinical Laboratory Standards Institute (2009). The NPs resistant strains were tested against ten different antibiotics (concentration ranges were expressed in μg/ml), including ampicillin (0.016–256), ampicillin-sulbactam (0.016–256), cefuroxime (0.016–256), tetracycline (0.016–256), doxycycline (0.016–256), ciprofloxacin (0.002–32), levofloxacin (0.002–32), trimethoprim- sulfamethoxazole (0.002–32), chloramphenicol (0.016–256), rifampin (0.016–256). In brief, strains of *Brucella* were plated on Mueller-Hinton agar plates (Sigma Aldrich, USA) and incubated at 37°C for 5–7 consecutive days. Approximately four to five colonies were placed into 5 ml of 0.9% NaCl solution in sterilized test tubes. Using the Sensititre nephelometer, the turbidity of the growing broth culture was adjusted to approximately 1 × 10^5^ KbE/ml. In a subsequent step, 11 ml of Mueller-Hinton broth was inoculated with 15 μl of modified tryptone soya broth (TSB), and 50 μl of this mixture was placed into each well of a microtitre plate. Incubation was carried out at 37°C for 24 hours after foil-wrapping the plates. A Sensititre reader (TREK Diagnostic Systems, UK) was used to read the plates.

### Assessment of NP cytotoxicity via MTT assay

The cytotoxicity of the SNPs and GNPs was examined *in vitro* using eel kidney (EK) cells rather than mammalian cells, as fish cell culture has a wider temperature range than mammalian cell culture. EK cells were grown at temperatures ranging from 15 to 37°C. We evaluated the cytotoxicity of the SNPs and GNPs *in vitro*. First, 100 μl of EK cells was laden in 96-well plates at a density of approximately 1.5×10^5^ cells/well in Leibowitz medium (Sigma-Aldrich, USA) supplemented with 5–10% foetal serum (ThermoFisher Scientific, USA) and antibiotic solution and then maintained at 37°C with 5% CO_2_ for one day. After incubation, the EK-1 cells were treated with 100 μl of three concentrations of each NP (12.5 μg/ml, 25 μg/ml and 50 μg/ml) and then kept at 37°C under 5% CO_2_ for a two-day period. The morphological features of the cells were then investigated under a microscope. Cells in medium without NPs were considered negative controls. Afterwards, 20 μl of 3-[4,5-dimethylthiazol-2-yl]-2,5-diphenyl tetrazolium bromide (MTT; 5 mg/ml) was added to every well. The plates were then incubated for 6 hours at 37°C, and the medium was discarded. Subsequently, the formazan crystals were dissolved in 100 μl of dimethyl sulfoxide (solubilization solution, Sigma–Aldrich). The absorbance value of each well was measured at 560 nm using a Multiskan™ FC Microplate Photometer (ThermoFisher Scientific, USA). Duplicate tests were carried out for each concentration and the control. After blank correction, the cell feasibility was calculated according to the formula % cell feasibility = (absorbance of NP-treated cells/absorbance of untreated cells) × 100.

### The toxic effects of SNPs and GNPs in experimental rats

In the present experiment, 100 male adult albino rats 50 to 80 days old with a body weight of 160±40 gm were used to examine the safety of *SNPs and GNPs in vivo*. Wire boxes were used to raise the rats with 5 rats in each cage. The rats were classified into 3 sets: S1, S2, and S3. The last set (S3) was considered the control set (20 rats), and these rats received salt solution (0.9%) during the experimental study. The other 2 sets, S1 and S2, were considered the investigational sets, which were further subdivided into 4 sets (10 rats/set) based on the experimental dose of NPs.

The rats were fed a normal diet under good hygienic measures. Free access to water was supplied to all sets of rats that were allowed free water, and all rats were reared at 25 ± 2°C. Fourteen days prior to the experimental study, the rats were acclimated to the investigation settings. The rats received 10-nm SNPs and 20-nm GNPs for 4 weeks. For oral administration of NPs at the study dosages of 0.5, 1, 2 and 4 mg/kg body weight, the equation below was applied: dose given = the experimental rat’s body mass × study dose/concentration of NPs × 1000. We used higher doses of NPs in this experiment than those used in the *in vitro* experiments for several reasons, including that the drug might not be well absorbed by the body or broken down quickly, or that only low concentrations of the drug might reach the affected region.

After 28 days, the rats were anaesthetized using ketamine-xylazine. The organs were then separated, washed with normal saline solution, and checked to examine their shape. Next, the organs were placed in formalin (10%) for stability and kept for histopathology. During the preparation of paraffin blocks, all tissue samples were processed using a tissue processor (Sakura Tissue Tek VIP E300). Slices with a thickness of 5 micrometres were cut, and staining was carried out with haematoxylin and eosin (H&E) to allow for histological inspection after embedding in paraffin. The effects of both NPs on rat tissue was confirmed by transmission electron microscopy (TEM). All procedures were conducted according to the National Institute of Health Guide for the Care and Use of Laboratory Animals and the Ethics Rules of the Laboratory Animal Care Center at Qassim University, KSA.

### Statistical analysis

The NP bactericidal impact data were imported into SPSS version 20.0, and this program was also used for all evaluations (Chicago, IL, USA).

## Results

### Detection of *Brucella* species

The Vitek 2 ID-GN card was used to biochemically identify 25 recovered isolates. The tested strains were reported as 14 *B*. *abortus* and 11 *B*. *melitensis* based on data analysis. The MALDI Biotyper (MBT) device was capable of recognizing all cultivated strains based on the gathered data. The MBT results were authenticated using real-time PCR. The target genes *BMEII0466* and *BruAb2 0168* were utilized to detect the *B*. *melitensis* and *B*. *abortus* strains, respectively. The base pairs of both genes were determined by running the qPCR outputs through the LabChip GXII Automatic gel apparatus, and the analysis indicated that *B*. *melitensis* had 112 bp and *B*. *abortus* had 222 bp. The MBT results were in complete agreement with the qPCR results; therefore, qPCR can now be utilized as an MBT validating procedure.

### The antimicrobial activity of SNPs and GNPs against *Brucella* species

The mean MIC values of the SNPs and GNPs against the *B*. *melitensis* and *B*. *abortus* isolates were determined using the broth microdilution technique and these data are reported in Table 2. For *B*. *melitensis*, the average MIC values for the 10-nm SNPs and 20-nm GNPs were 22.43±2.32 and 13.56±1.22 g/ml, respectively. For *B*. *abortus*, the mean MIC values were 18.77±1.33 and 12.45±1.59 g/ml, respectively. The range of MICs for the 10-nm SNPs varied from 12.5 to 25 μg/ml for *B*. *melitensis* and 6.25 to 50 μg/ml for *B*. *abortus* strains, whereas the MIC range for the 20-nm GNPs was 6.25–50 μg/ml for both *Brucella* species. Moreover, the MIC_90_ values for each NP were 50 μg/ml for *B*. *melitensis* and 25 μg/ml for *B*. *abortus*. These results show that almost all of the *Brucella* strains were vulnerable to the examined NPs and that only small amounts of NPs were required to destroy the *Brucella* isolates.

**Table 2:** MICs (μg/ml) for 10-nm SNPs and 20-nm GNPs against multidrug-resistant *B*. *melitensis* and *B*. *abortus* strains.

Nanosized particle	*B*. *melitensis*				*B*. *abortus*			
	Mean MIC	MIC range	MIC_50_	MIC_90_	Mean MIC	MIC range	MIC_50_	MIC_90_
**10-nm S**	22.43±2.32	12.5–100	12.5	50	18.77±1.33	6.25–50	12.5	50
**20-nm G**	13.56±1.22	6.25–50	6.25	50	12.45±1.59	6.25–50	6.25	25

**Fig 2:**
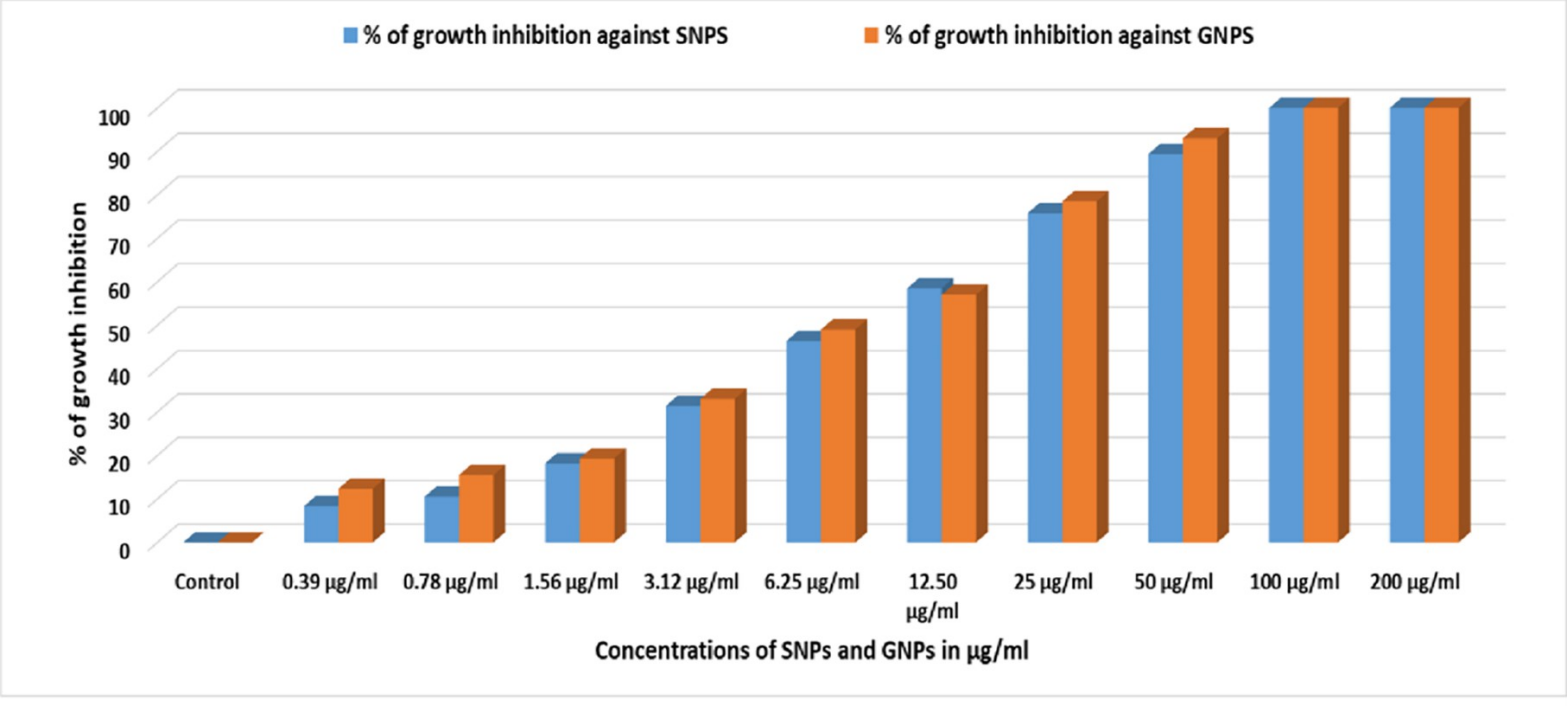
*In vitro* antibacterial activity of SNPs and GNPs against *Brucella* species. The graph shows the % growth inhibition vs. concentration for 10-nm SNPs and 20-nm GNPs.

The *in vitro* antimicrobial efficacy of the 10-nm SNPs and 20-nm GNPs was tested utilizing turbidimetric growth interpretation over a range of nanomaterial concentrations from 0.39 to 200 μg/ml (Fig 2). An average percent growth inhibition plot was constructed over the entire range of concentrations by plotting growth inhibition as a function of NP concentration, and the horizontal coefficient of determination between the two variables was calculated.

Additionally, we calculated the minimal bacterial concentration (MBC) for both types of NPs and discovered that at doses of 100 μg/ml and 200 μg/ml, all *Brucella* strains were totally suppressed compared to concentrations of 0.39–50 μg/ml.

### The development of resistance of *Brucella* species to SNPs and GNPs

To determine whether NP resistance may be acquired in *Brucella* species after a long time, we cultured 22 *Brucella* strains (10 *B*. *melitensis* and 12 *B*. *abortus*) with low doses of SNPs and GNPs. According to our findings, none of the *Brucella* strains elicited resistance to the 10-nm SNPs after 10^th^ stable passage, with the exception of 5 strains of *B*. *melitensis* and 7 strains of *B*. *abortus* which demonstrated an increase in the MICs of the 10-nm SNPs. Likewise, the 20-nm GNPs had higher MICs for six *B*. *melitensis* and six *B*. *abortus* strains.

### The potential resistance to antibiotics after treatment with NPs

Consistent with the previously established methodologies for inactive microorganisms (*Haemophilus* species), *Brucella* breakpoints against antibacterial drugs under study has been identified [52]. Therefore, we tested the antimicrobial resistance of 13 *Brucella* strains, including 7 strains that had received lengthy exposure to 10-nm SNPs and 6 strains that had received extended exposure to GNPs. Except for one (7.69%) of the *Brucella* strains that had been formerly resistant to the SNPs and 20-nm GNPs and exhibited significant resistance to rifampin and ampicillin, all isolates demonstrated greater sensitivity to all of the tested antimicrobial drugs. Additionally, only two strains (15.38%) displayed considerable stable resistance against trimethoprim-sulfamethoxazole. After the tenth sustained exposure, the average MIC values for rifampin, ampicillin and trimethoprim-sulfamethoxazole were 1.52, 1.11 and 1.29 mg/L, respectively; overall, no tolerance to the spectrum of the drugs utilized in this investigation was found.

### *In vitro* cytotoxicity of the SNPs and GNPs at various concentrations

SNPs and GNPs have a wide range of persistence in EK-1 cells. Comparing the viabilities of the cells treated with 12.5 μg/ml SNPs and GNPs with that of untreated control cells, we did not find a significant difference (P ≥ 0.05). After incubating the cells with 25 and 50 μg/ml SNPs, cell viability was 70.32 ± 16.54% and 52.78 ± 12.43%, respectively. Cell viability was also measured after the cells were incubated with 25 and 50 μg/ml SNPs, showing 73.45 ± 22.64% and 59.23 ± 14.58% viability, respectively (Table 3).

**Table 3:** Variations in the viability of EK-1 cells treated with different concentrations of SNPs and GNPs (0, 12.5, 25, 50 μg/ml). The values are exhibited as the mean ± standard deviation.

Concentration (μg/ml)	Cell viability (%) after treatment with SNPs	Cell viability (%) after treatment with GNPs
0.0	99.92 ± 12.67	100.32 ± 15.23
12.5	88.34 ± 10.34	92.43 ±12.76
25	70.32 ± 16.54	73.45 ± 22.64
50	52.78 ± 12.43	59.23 ± 14.58

### *In vivo* toxicity of the SNPs and GNPs in experimental rats

#### Rat morphological alterations

The hair colour of the experimental rats given a 2 mg/kg dose of 10-nm SNPs showed some visible alterations. Furthermore, once the brains, livers, kidneys, and hearts of the S1 and S2 groups of rats were compared with those of the control rats, certain external impairments were detected. The S2 group, which was given 20-nm GNPs, displayed important morphological alterations in the colouring and shrinkage of the liver. The following formula for the spleen index (SI) was used to determine the extent of the alterations generated by SNPs and GNPs: SI = body mass of the investigated organ/body mass of the investigated animal/body mass of the untreated animal/body mass of the untreated animal. In the sets of rats, the average SI score was 0.96 ± 0.01, which was a bit less than the rats in the control group (0.99 ± 0. 021). NP doses ≥ 2 mg/kg are associated with decreased splenic index. The shapes of the various organs of the healthy control rats were essentially identical; nevertheless, the colouration of the spleens in S1 and S2 animals changed dramatically to a metallic brown colour. These data suggest that the spleen is among the organs targeted by these NPs. There were no additional tissues that revealed any significant morphological alterations.

#### Rat histopathological alterations

**Fig 3:**
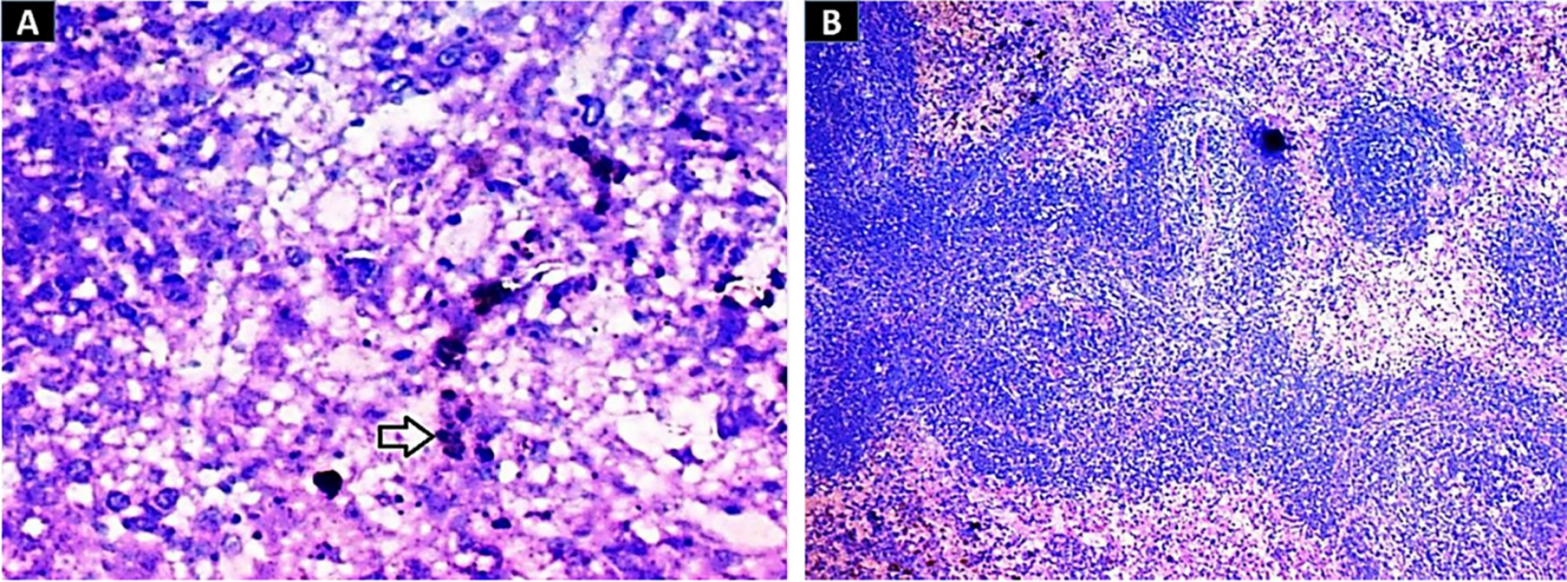
Images of splenic tissue stained with H&E (400×). (A) A splenic tissue section with noticeable stain accumulation as evidenced by the black arrow in the group treated with 10-nm SNPs (2 mg/kg). (B) Splenic tissue segment with underdeveloped follicles, bleeding, and hyperpigmentation induced by 20-nm GNPs.

**Fig 4:**
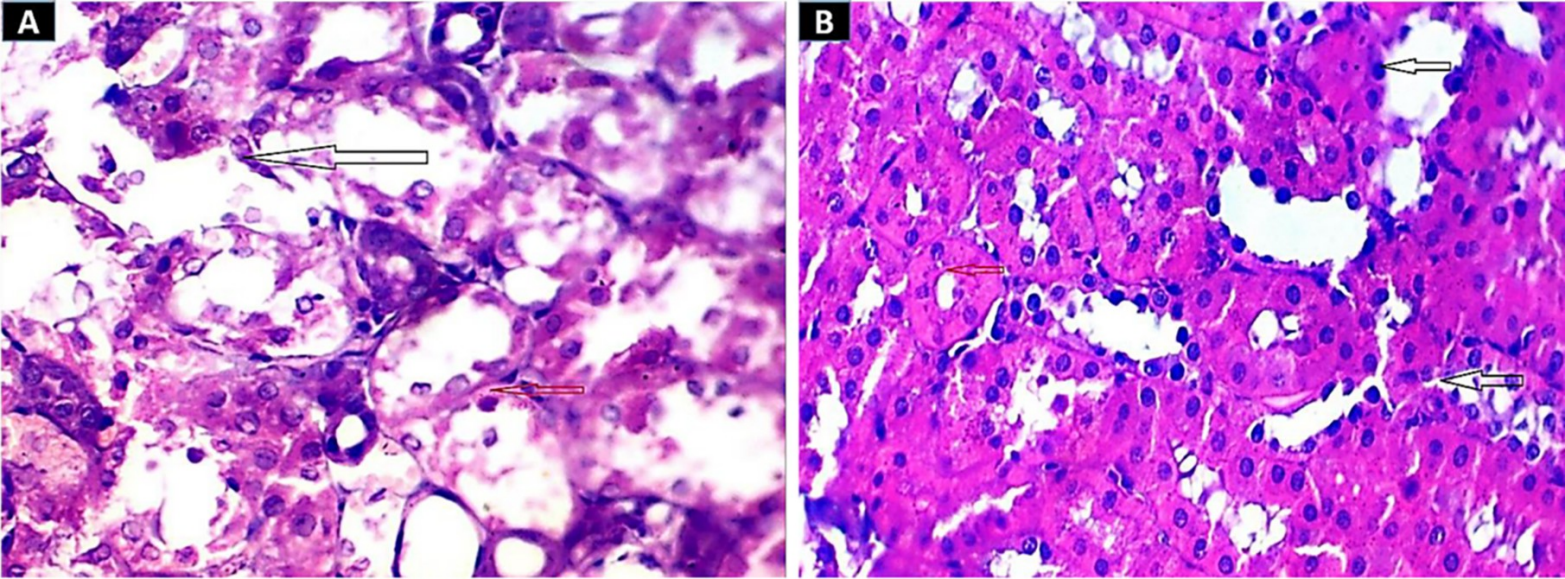
H&E staining images (400×) of kidney tissue. (A) In the 10-nm SNP-treated group, the black arrows indicate marked tubular necrosis, and the red arrow indicates colouration of the nanoparticles. (B) In the group treated with 20-nm GNPs, hazy swelling was seen.

**Fig 5:**
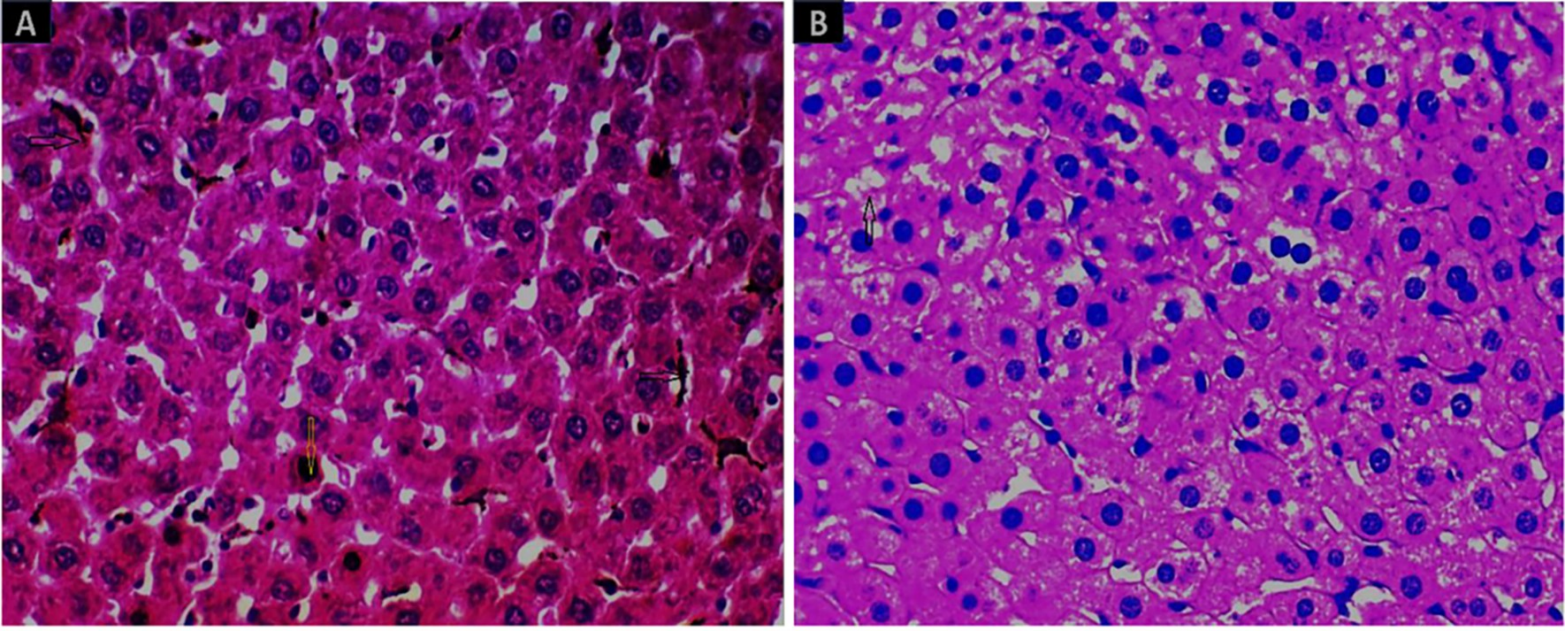
H&E staining images of hepatic tissue (400×). (A) Apoptotic cell death is indicated by the dark arrows, while nanoparticle hyperpigmentation in the hepatocytes from the rats treated with 10-nm S NPs set is indicated by the yellow arrow. (B) Liver tissue section with liquefactive degradation (dark arrow) caused by 20-nm GNP treatment.

**Fig 6:**
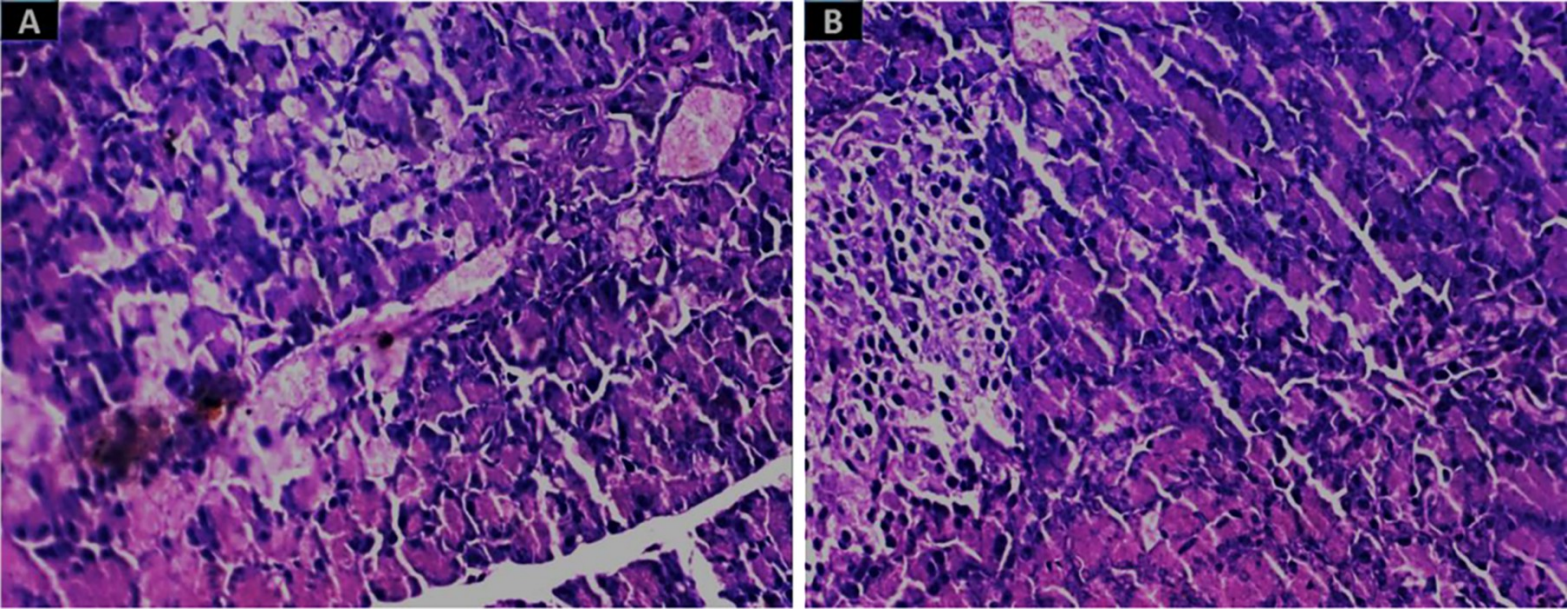
Images of pancreatic tissue stained with H&E (400× magnification). (A) Substantial hydropic degradation and colouration were observed in the 10-nm SNP-treated group. (B) Interstitial septa thickening and pigment precipitation were seen in the group treated with 20-nm GNPs.

**Fig 7:**
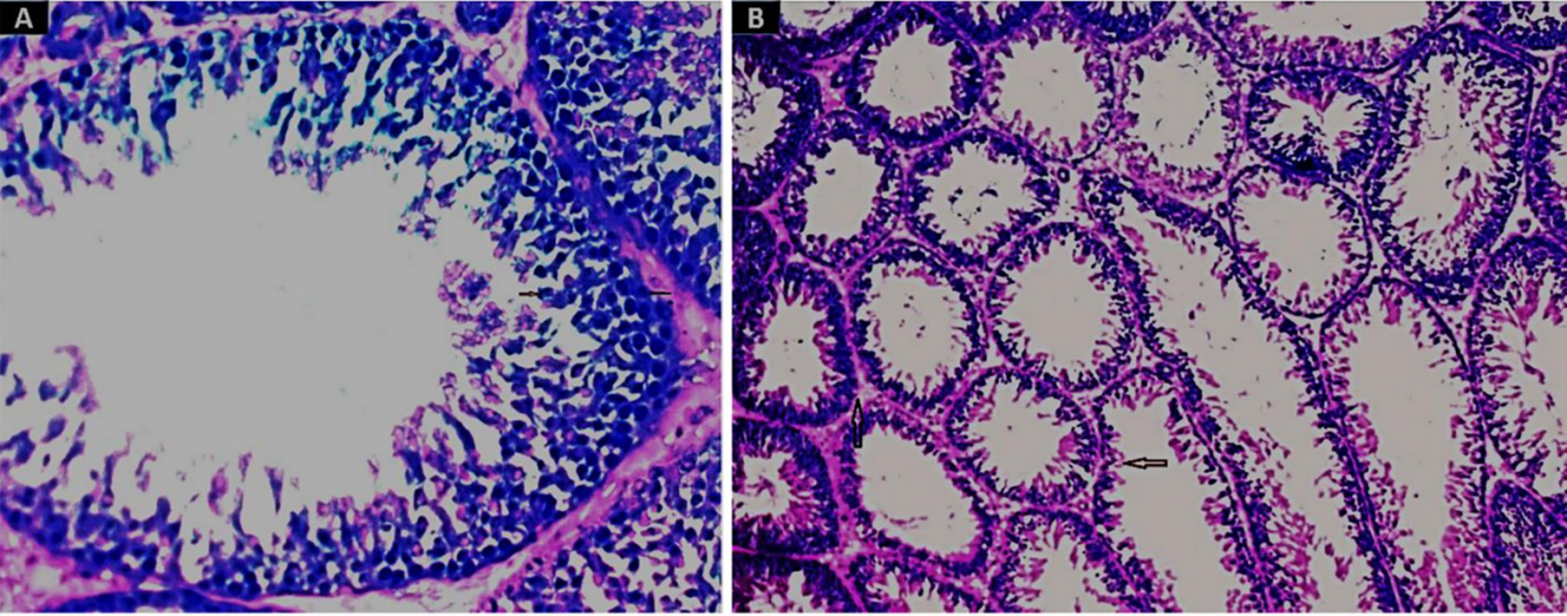
H&E-stained images of testicular tissue (400× magnification). The black arrows indicate the thickened basement membrane and considerable hypospermatogenesis in the groups administered 2 mg/kg of SNPs (A) and GNPs (B).

**Fig 8:**
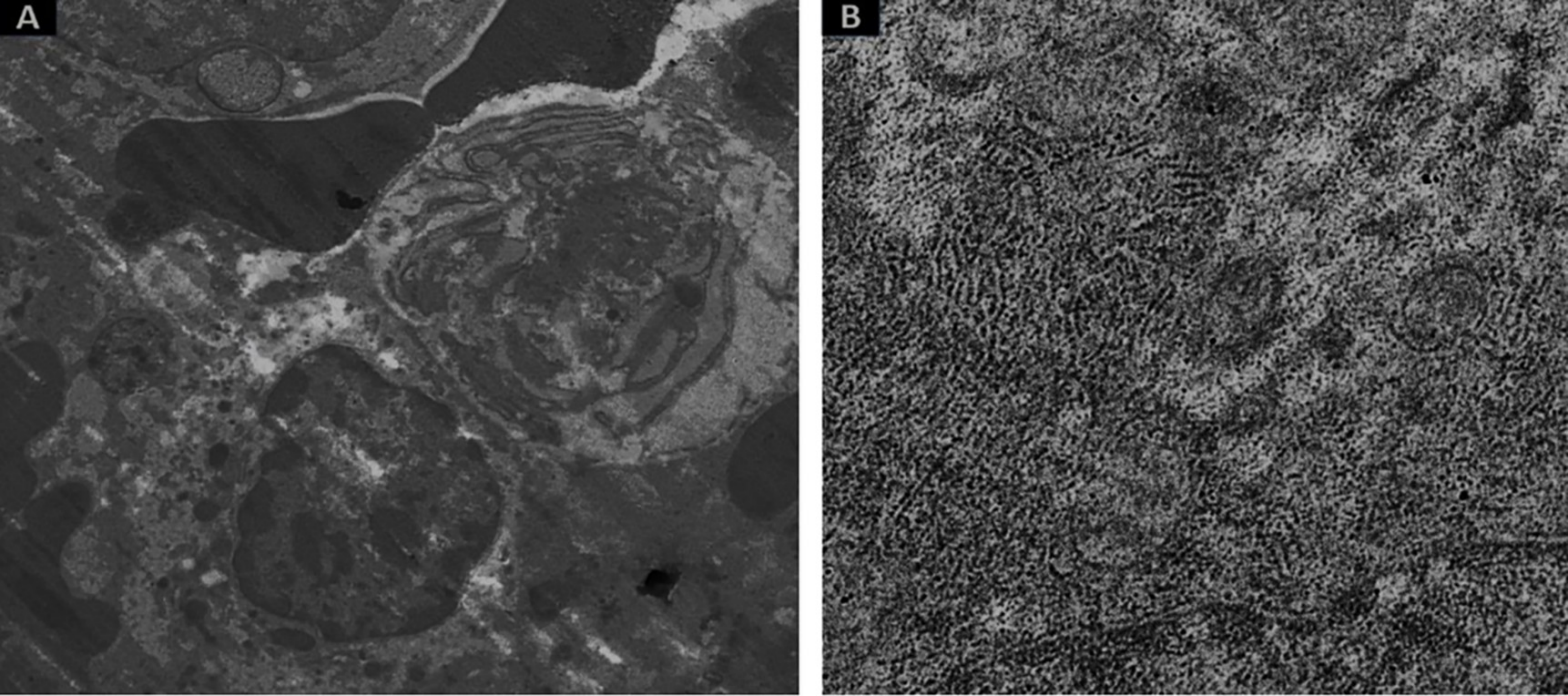
Images of splenic and hepatic tissues taken with a transmission electron microscope. (A) Two electron-dense GNPs are illustrated inside two splenic macrophage lysosomes. (B) Hepatocytic cells from the group treated with 10-nm SNPs showed the accumulation of NPs in Kupffer cells.

After 30 days of oral NP administration to rats, the negative consequences of the NPs were examined. Histopathological examination of the various investigated organs demonstrated no negative impacts after treatment with the experimental doses of 0.25 to 1 mg of 10-nm SNPs and 20-nm GNPs. 10-nm SNPs and 20-nm GNPs at 2 mg/kg, on the other hand, were harmful to all of the tissues investigated. The histopathological analysis revealed that NP deposition and apoptosis induction occurred in nearly all investigated organ systems (Figs 4–8), and in particular in the splenic tissue segment, which displayed pigment accumulation as indicated by the black arrow in Fig 3A for the group treated with 2 mg of 10-nm SNPs and underdeveloped follicles, bleeding, and hyperpigmentation in the group treated with 2 mg of GNPs (Fig 3B).

The renal tissue of the mice in the group treated with SNPs showed severe tubular deterioration, as indicated by the black arrows in Fig 4A and the red arrow denotes nanoparticle pigmentation, whereas the mice treated with GNPs showed hazy swelling (Fig 4B). In the liver sections, apoptotic cell death and NP hyperpigmentation of hepatocytes were identified in the SNP-treated group (Fig 5A), and liquefactive necrosis was observed in the GNP-treated rats (Fig 5B). Furthermore, the pancreatic tissue images revealed substantial hydropic degeneration in the mice administered SNPs (Fig 6A), and interstitial septa thickening and pigment precipitation were seen in the group of mice treated with GNPs (Fig 6B). In the testicular tissue sections, thickening of the basement membrane and considerable hypospermatogenesis were observed in both the SNP- (Fig 7A) and GNP-treated (Fig 7B) groups. Images of the splenic and hepatic tissues taken with a transmission electron microscope displayed the presence of SNPs inside two splenic macrophage lysosomes (Fig 8A), and NP accumulation was observed within Kupffer cells in the group treated with GNPs (Fig 8B).

## Discussion

One of the leading major problems facing the health care system worldwide is communicable diseases. Both the WHO and the CDC have expressed grave concerns over the consistent increase in antibiotic resistance among microorganisms. Antibiotic resistance is therefore one of the most significant global health security challenges. Antibiotic resistance is linked to the emergence of antimicrobial resistance, and there are no new antimicrobial drugs available [1, 22]. Brucellosis is still a common illness worldwide, a major public health concern, and an economic burden in agriculture. Despite the availability of efficient vaccines for livestock, treating brucellosis in both animals and people remains difficult [65].

Thus, the development of new therapies is necessary to address chronic bacterial diseases, especially intracellular infections, which are extremely difficult to treat. Using NPs makes it possible to overcome current obstacles in the treatment of intracellular bacteria, such as *B*. *melitensis*, leveraging the size and charge of cellular membranes [16]. NPs, especially SNPs, are currently considered to be the next generation of antibiotics due to their inhibitory effects against various pathogens. Based on our findings, the mean MIC values of 10-nm SNPs against *B*. *melitensis* and *B*. *abortus* were 22.43 ± 2.32 and 18.77 ± 1.33 μg/ml, respectively. Alizadeh et al. [66] obtained similar results, determining that the MIC and MBC of SNPs against *B*. *abortus* 544 were 6 μg/ml and 8 μg/ml, respectively. Several experiments have shown that SNP size drastically affects their efficacy [67, 68]. The best antimicrobial performance was reported for SNPs smaller than 30 nm against gram-positive bacteria (e.g., *Staphylococcus aureus*) and gram-negative bacteria (e.g., *Klebsiella pneumonia*) [69]. The interactions of these SNPs with membranes and their possible disruption, which could harm cells, are more notable when the particles have a smaller diameter and a positive zeta potential.

SNPs may possess strong antibacterial properties due to their capability to physically interact with bacterial surfaces. This is especially crucial in the case of gram-negative bacteria, where SNP adherence and deposition to the bacterial surface have been reported in multiple investigations [23]. Numerous studies have shown that SNPs damage cell membranes, altering the bacterial structure, making them more prone to producing infections [22, 70, 71]. The effects are also strongly influenced by the surface area, morphology, and adsorbent dose of the particles [23, 71, 72]. One study on the gram-negative bacteria *Escherichia coli* revealed that SNP build-up on the cell membrane causes holes in the bilayer, resulting in an increased diffusion coefficient and ultimately bacterial cell death [73].

In the biomedical field, GNPs have been recognized as novel metal NPs. GNPs can be differentiated from other metal NPs and typical metal materials. GNPs have been shown to be effective against a wide variety of microorganisms, and studies on their antimicrobial properties have become increasingly popular in recent years [74]. In the current study, the MIC values for 20-nm GNPs were 13.56 ± 1.22 μg/ml for *B*. *melitensis* and 12.45 ± 1.59 μg/ml for *B*. *abortus* [75]. It was previously reported that GNPs at concentrations of 0.1 and 1.5 μg/mL were able to kill 90% of *Escherichia coli* as observed by the transmission electron microscopy images that showed cell lysis. Tian and colleagues [74] studied the antibacterial activity of GNPs with sizes ranging from 7 to 34 nm against *Staphylococcus aureus* and *Bacillus subtilis* and found that the MIC value was 7.56 μg/ml for both bacteria. The ability of GNPs to inhibit the activity of the bacterial cell membrane and increase the calcium ion concentration in the cytoplasm explains their potent antibacterial activity [74]. According to Lee et al. [76], bacterial DNA damage caused apoptosis-like cell death after *Escherichia coli* were exposed to GNPs.

Following the development of resistance in the 22 *Brucella* strains tested here, we observed that the 10-nm SNPs caused resistance in only 7/22 (31.82%) of the tested strains. After ten passages, the 20-nm GNPs were also able to generate resistance in 6/22 (27.27%) *Brucella* strains. All Ag- and GNP-treated *Brucella* strains from the MIC study showed resistance stability after the tenth sustained passage in NP-independent media. Regardless of how effective an antimicrobial agent is; most microorganisms will find a way to defeat it. The flexibility of the bacterial genome, as well as the selective pressure by most nanomaterials, frequently promote the emergence of resistant bacteria [77]. Thus, despite the impact of several kinds of NPs against bacteria, microbial resistance to nanomaterials has been documented. According to Mba et al. [77] and Niño-Martínez et al. [78], resistance mechanisms identified in bacteria include efflux pumps, electrostatic repulsion, morphological changes, and mutagenesis. The exposure of gram-negative bacteria (e.g., *Pseudomonas aeruginosa*) to NPs changed the unsaturated fatty acid content in the cell membrane according to a prior study by Hachicho et al. [79]. The membrane fluidity changes as a result of this shift, making it difficult for NPs to pass through. Moreover, Graves Jr et al. [80] found that exposing microorganisms to sublethal doses of NPs can aid in the establishment of resistance through disorders that cause overexpression and reduced expression of several genes.

The majority of strains in the current investigation remained susceptible to both types of NPs after the 10^th^ stable passage. This result can be explained by the NPs having distinct diameters of less than 100 nm, which typically leads to the destruction of microbes through a particular mode of action. Because of their unusually small size, these NPs have a variety of unique properties, including outstanding contact with microbial cells due to a higher surface-to-volume ratio, as well as adaptable and manageable utilization [22, 81]. These characteristics make it extremely difficult for pathogens to establish resistance to NPs. Several antibiotics were tested against *Brucella* strains that were both SNP- and GNP-resistant in this study. We did not find a relationship between NP resistance and antimicrobial resistance, as previously reported; nevertheless, Elbehiry et al. [22], El Behiry et al. [55] and Karatzas et al. [82] investigated the resistance between antiseptics and antimicrobial drugs and discovered that bacteria resistant to antiseptics were likewise resistant to antimicrobials. The lack of cross-resistance in our work shows that the pathways that contribute to NP resistance are distinct from those that lead to resistance to antibiotics.

The toxicity of NPs has attracted much attention, as it constitutes a potential health and ecological concern. NP toxicity indicates a relationship between the physicochemical properties of the NPs and their biological consequences from a scientific standpoint [83]. After orally dosing rats with different concentrations of NPs for 28 days, we studied the toxic effects of 10-nm SNPs and 20-nm GNPs in several organs (spleen, kidney, liver, pancreas, and testes). SNPs and GNPs were not harmful to the splenic, renal, hepatic, pancreatic, and testicular tissues at 0.25, 0.5, or 1 mg/kg according to histological examinations. On the other hand, the greater dose (2 mg/kg) of NPs severely harmed all of the tissues studied, causing apoptosis, NP accumulation, cell death, and tissue destruction. Similarly, Elbehiry et al. [22] studied the toxic effects of 10-nm SNPs and 20-nm GNPs in rats after oral dosing at 0.25, 0.5, 1, and 2 mg/kg and found that 2 mg/kg resulted in impairment of hepatic, renal, splenic, and lung tissues. Sardari et al. [60] also found similar effects in the hepatic, splenic and renal tissues of experimental rats when they tested the hazardous effects of larger 70-nm SNPs at various doses. They also discovered that at 2 mg/kg, these SNPs caused tissue injury, internal bleeding, cell destruction, and apoptosis. In other studies, Park et al. [84] examined the toxicity of 22 nm, 71 nm, and 323 nm SNPs in rats. Based on the observations of the researcher, exposure to 323-nm SNPs did not cause considerable alterations in hepatic, renal, or splenic tissues; however, exposure to 22-nm or 71-nm SNPs led to NP uptake from the intestinal mucosa.

Park et al. [84] and Weldon et al. [85] analysed the liver damage caused by long-term oral NP treatment in rats administered 0.25 to 1 mg/kg 42-nm SNPs. The cell membrane damage produced large quantities of oxygen radicals, and the consequential organ damage was caused by this factor. NP clearance in the kidney is highly dependent on molecule size [86]. NPs <6 nm are completely filtered, while those ˃8 nm are rarely filtered [22, 87]. We discovered that when rats were given 2 mg/kg 10-nm SNPs and 20-nm GNPs, they had severe tubular atrophy of the renal tissue. The aggregation of SNPs and GNPs induced tissue deterioration, which eventually caused tubular shrinkage. Moreover, another study discovered that silver ions are widely disseminated throughout the body and eventually clump together in different organs, including hepatic, splenic, and renal tissues [22]. Silver and gold ions are suspected to become entangled with thiol groups in hepatic cells, stimulate reduction responses, transport glutathione to the bile duct, and decrease glutathione levels in rats given SNPs or GNPs based on analyses of the splenic, hepatic, renal, pancreatic, and testicular tissues. However, more research on the effects of NPs at varying concentrations and in different forms with different dimensions is necessary to guarantee the safety and utility of this revolutionary technology.

## Conclusions

As a result of our investigations, it was found that *Brucella* strains isolated from both humans and animals suffering from brucellosis are extremely sensitive to SNPs and GNPs. After many stable passages in free media, both *B*. *melitensis* and *B*. *abortus* exhibited similar little resistance to SNPs and GNPs. The majority of *Brucella* strains examined did not show any cross-resistance between the NPs and antimicrobial drugs frequently utilized for the treatment of brucellosis. SNPs and GNPs are also not hazardous at low levels according to histological analyses of numerous organs from the treated rats. The greater dose (2 mg/kg) of NPs, on the other hand, caused substantial deficits in all of the tissues evaluated.

### Limitations of the study

The lack of antimicrobial screening for different doses of SNPs and GNPs in infected experimental animals is considered to be one of the study’s limitations. Additional studies are needed to evaluate the *in vivo* antimicrobial effects of NPs.

## Supporting information

S1 File Springer nature certificate (English editing). (PDF)

## References

[1] Baptista PV, McCusker MP, Carvalho A, Ferreira DA, Mohan NM, Martins M, et al. Nano-strategies to fight multidrug resistant bacteria—“A Battle of the Titans”. Frontiers in microbiology. 2018; 9: 1441. doi: 10.3389/fmicb.2018.01441 30013539 PMC6036605

[2] Thorpe KE, Joski P, Johnston KJ. Antibiotic-resistant infection treatment costs have doubled since 2002, now exceeding $2 billion annually. Health Affairs. 2018; 37(4): 662–669. doi: 10.1377/hlthaff.2017.1153 29561692

[3] Morris S, Cerceo E. Trends, epidemiology, and management of multi-drug resistant gram-negative bacterial infections in the hospitalized setting. Antibiotics. 2020; 9(4): 196. 10.3390/antibiotics9040196 PMC7235729 32326058

[4] Darabpour E, Bavi AP, Motamedi H, Nejad SM. Antibacterial activity of different parts of Peganum harmala L. growing in Iran against multi-drug resistant bacteria. EXCLI journal. 2011; 10: 252. 29033706 PMC5611620

[5] Guglielmi P, Pontecorvi V, Rotondi G. Natural compounds and extracts as novel antimicrobial agents. Expert Opinion on Therapeutic Patents. 2020; 30(12): 949–962. doi: 10.1080/13543776.2020.1853101 33203288

[6] Kirk MD, Pires SM, Black RE, Caipo M, Crump JA, Devleesschauwer B, et al. World Health Organization estimates of the global and regional disease burden of 22 foodborne bacterial, protozoal, and viral diseases, 2010: a data synthesis. PLoS medicine. 2015; 12(12): e1001921. doi: 10.1371/journal.pmed.1001921 26633831 PMC4668831

[7] Raghava S, Mbae KM, Umesha S. Green synthesis of silver nanoparticles by Rivina humilis leaf extract to tackle growth of *Brucella* species and other perilous pathogens. Saudi Journal of Biological Sciences. 2021; 28(1): 495–503. doi: 10.1016/j.sjbs.2020.10.034 33424332 PMC7785426

[8] Doganay M, Aygen B. Human brucellosis: an overview. International journal of infectious diseases. 2003; 7(3): 173–182.

[9] Grilló MJ, Manterola L, De Miguel MJ, Muñoz PM, Blasco JM, Moriyón I, et al. Increases of efficacy as vaccine against *Brucella abortus* infection in mice by simultaneous inoculation with avirulent smooth bvrS/bvrR and rough wbkA mutants. Vaccine. 2006; 24(15): 2910–2916. doi: 10.1016/j.vaccine.2005.12.038 16439039

[10] Azad ZM, Moravej H, Fasihi-Ramandi M, Masjedian F, Nazari R, Mirnejad R, et al. *In vitro* synergistic effects of a short cationic peptide and clinically used antibiotics against drug-resistant isolates of *Brucella melitensis*. Journal of medical microbiology. 2017; 66(7): 919–926. doi: 10.1099/jmm.0.000524 28699872

[11] Azam S, Rao SB, Jakka P, NarasimhaRao V, Bhargavi B, Gupta VK, et al. Genetic characterization and comparative genome analysis of *Brucella melitensis* Isolates from India. International journal of genomics. 2016. doi: 10.1155/2016/3034756 27525259 PMC4976149

[12] Gamazo C, Concepcion Lecaroz M, Prior S, Isabel Vitas A, Campanero MA, Irache JM, et al. Chemical and biological factors in the control of Brucella and brucellosis. Current Drug Delivery. 2006; 3(4): 359–65. doi: 10.2174/156720106778559038 17076637

[13] Solís García del Pozo J, Solera J. Systematic review and meta-analysis of randomized clinical trials in the treatment of human brucellosis. PloS one. 2012; 7(2): e32090. doi: 10.1371/journal.pone.0032090 22393379 PMC3290537

[14] Al Barraq AA, Makeen HA, Menachery SJ. Pediatric brucellosis: A short review. Saudi Journal for Health Sciences. 2018; 7(1): 1.

[15] Ersoy Y, Sonmez E, Tevfik MR, But AD. Comparison of three different combination therapies in the treatment of human brucellosis. Tropical doctor. 2005; 35(4): 210–2. doi: 10.1258/004947505774938765 16354469

[16] Lueth P, Haughney SL, Binnebose AM, Mullis AS, Peroutka-Bigus N, Narasimhan B, et al. Nanotherapeutic provides dose sparing and improved antimicrobial activity against Brucella melitensis infections. Journal of Controlled Release. 2019; 294: 288–297. doi: 10.1016/j.jconrel.2018.12.024 30572034

[17] Imbuluzqueta E, Gamazo C, Lana H, Campanero MÁ, Salas D, Gil AG, et al. Hydrophobic gentamicin-loaded nanoparticles are effective against Brucella melitensis infection in mice. Antimicrobial agents and chemotherapy. 2013; 57(7): 3326–33. doi: 10.1128/AAC.00378-13 23650167 PMC3697350

[18] Vilchis-Nestor AR, Sánchez-Mendieta V, Camacho-López MA, Gómez-Espinosa RM, Camacho-López MA, Arenas-Alatorre JA. Solventless synthesis and optical properties of Au and Ag nanoparticles using Camellia sinensis extract. Materials letters. 2008; 62(17–18): 3103–3105.

[19] Wang L, Hu C, Shao L. The antimicrobial activity of nanoparticles: present situation and prospects for the future. International journal of nanomedicine. 2017; 12: 1227. doi: 10.2147/IJN.S121956 28243086 PMC5317269

[20] Sadeghi B, Jamali M, Kia S, Amini NA, Ghafari S. Synthesis and characterization of silver nanoparticles for antibacterial activity. 2010; 119–124.

[21] Boschi F, De Sanctis F. Overview of the optical properties of fluorescent nanoparticles for optical imaging. European Journal of Histochemistry. 2017; 61(3). doi: 10.4081/ejh.2017.2830 29046056 PMC5579469

[22] Elbehiry A, Al‐Dubaib M, Marzouk E, Moussa IM. Antibacterial effects and resistance induction of silver and gold nanoparticles against *Staphylococcus aureus*‐induced mastitis and the potential toxicity in rats. MicrobiologyOpen. 2019; 8(4): e00698. doi: 10.1002/mbo3.698 30079629 PMC6460268

[23] Franci G, Falanga A, Galdiero S, Palomba L, Rai M, Morelli G, et al. Silver nanoparticles as potential antibacterial agents. Molecules. 2015; 20(5): 8856–8874. doi: 10.3390/molecules20058856 25993417 PMC6272636

[24] Salomoni R, Léo P, Rodrigues MFA. Antibacterial activity of silver nanoparticles (AgNPs) in *Staphylococcus aureus* and cytotoxicity effect in mammalian cells. Substance. 2015; 17: 18.

[25] Castro-Mayorga JL, Freitas F, Reis M, Prieto MA, Lagaron JM. Biosynthesis of silver nanoparticles and polyhydroxybutyrate nanocomposites of interest in antimicrobial applications. International journal of biological macromolecules. 2018; 108: 426–435. doi: 10.1016/j.ijbiomac.2017.12.007 29217186

[26] Zannella C, Shinde S, Vitiello M, Falanga A, Galdiero E, Fahmi A, et al. Antibacterial activity of indolicidin-coated silver nanoparticles in oral disease. Applied Sciences. 2020; 10(5): 1837.

[27] Panáček A, Smékalová M, Kilianová M, Prucek R, Bogdanová K, Večeřová R, et al. Strong and nonspecific synergistic antibacterial efficiency of antibiotics combined with silver nanoparticles at very low concentrations showing no cytotoxic effect. Molecules. 2016; 21(1); 26. 10.3390/molecules21010026 PMC6273824 26729075

[28] Liao C, Li Y, Tjong SC. Bactericidal and cytotoxic properties of silver nanoparticles. International journal of molecular sciences. 2019; 20(2): 449. doi: 10.3390/ijms20020449 30669621 PMC6359645

[29] White RJ. An historical overview of the use of silver in wound management. British Journal of Community Nursing 6(Sup1). 2001; 3–8.

[30] Taraszkiewicz A, Fila G, Grinholc M, Nakonieczna J. Innovative strategies to overcome biofilm resistance. BioMed research international. 2013. doi: 10.1155/2013/150653 23509680 PMC3591221

[31] Ahamed M, AlSalhi MS, Siddiqui MKJ. Silver nanoparticle applications and human health. Clinica chimica acta. 2010; 411(23–24): 1841–1848. doi: 10.1016/j.cca.2010.08.016 20719239

[32] Zhang XF, Li Z.G, Shen W, Gurunathan S. Silver nanoparticles: synthesis, characterization, properties, applications, and therapeutic approaches. International journal of molecular sciences. 2016; 17(9): 1534. 10.3390/ijms17091534 PMC5037809 27649147

[33] Skóra B, Krajewska U, Nowak A, Dziedzic A, Barylyak A, Kus-Liśkiewicz M. Noncytotoxic silver nanoparticles as a new antimicrobial strategy. Scientific Reports. 2021; 11(1): 1–13. 34188097 10.1038/s41598-021-92812-w PMC8242066

[34] Ahmad A, Wei Y, Syed F, Tahir K, Rehman AU, Khan A, et al. The effects of bacteria-nanoparticles interface on the antibacterial activity of green synthesized silver nanoparticles. Microbial pathogenesis. 2017; 102: 133–142. doi: 10.1016/j.micpath.2016.11.030 27916692

[35] Mahmoudi M, Serpooshan V. Silver-coated engineered magnetic nanoparticles are promising for the success in the fight against antibacterial resistance threat. ACS nano. 2012; 6(3): 2656–2664. doi: 10.1021/nn300042m 22397679

[36] Rai MK, Deshmukh SD, Ingle AP, Gade AK. Silver nanoparticles: the powerful nanoweapon against multidrug‐resistant bacteria. Journal of applied microbiology. 2012; 112(5): 841–852. doi: 10.1111/j.1365-2672.2012.05253.x 22324439

[37] Khan MH, Unnikrishnan S, Ramalingam K. Bactericidal potential of silver-tolerant bacteria derived silver nanoparticles against multi drug resistant ESKAPE pathogens. Biocatalysis and Agricultural Biotechnology. 2019; 18: 100939.

[38] Mulani MS, Kamble E., Kumka SN., Tawre MS, Pardesi KR. Emerging strategies to combat ESKAPE pathogens in the era of antimicrobial resistance: a review. Frontiers in microbiology. 2019; 10: 539. doi: 10.3389/fmicb.2019.00539 30988669 PMC6452778

[39] Sreekanth TVM, Nagajyothi PC, Lee KD. Biosynthesis of gold nanoparticles and their antimicrobial activity and cytotoxicity. Advanced Science Letters. 2012; 6(1); 63–69.

[40] Wang J, Li Q, Xue J, Chen W, Zhang R, Xing D. Shape matters: Morphologically biomimetic particles for improved drug delivery. Chemical Engineering Journal. 2021; 410: 127849.

[41] Mihai S, Malaisteanu M. Size-dependent antibacterial of gold colloids. Revista de Chimie-Bucharest. 2013; 64: 105–107.

[42] Zhang Y, Shareena Dasari TP, Deng H, Yu H. Antimicrobial activity of gold nanoparticles and ionic gold. Journal of Environmental Science and Health, Part C. 2015; 33(3): 286–327. doi: 10.1080/10590501.2015.1055161 26072980

[43] MubarakAli D, Thajuddin N, Jeganathan K, Gunasekaran M. Plant extract mediated synthesis of silver and gold nanoparticles and its antibacterial activity against clinically isolated pathogens. Colloids and Surfaces B: Biointerfaces. 2011; 85(2): 360–365. doi: 10.1016/j.colsurfb.2011.03.009 21466948

[44] Shamaila S, Zafar, Riaz S, Sharif R, Nazir J, Naseem S Gold nanoparticles: an efficient antimicrobial agent against enteric bacterial human pathogen. Nanomaterials. 2016; 6(4): 71. doi: 10.3390/nano6040071 28335198 PMC5302575

[45] Yousefi M, Dadashpour M, Hejazi M, Hasanzadeh M, Behnam B, de la Guardia M, et al. Anti-bacterial activity of graphene oxide as a new weapon nanomaterial to combat multidrug-resistance bacteria. Materials Science and Engineering: C. 2017; 74: 568–581. doi: 10.1016/j.msec.2016.12.125 28254332

[46] Bhosale S, Ekambe P, Bhoraskar S, Mathe VL. Effect of surface properties of NiFe2O4 nanoparticles synthesized by dc thermal plasma route on antimicrobial activity. Applied Surface Science. 2018; 441: 724–733.

[47] Oluwatoyin F, Gabriel O, Olubunmi A, Olanike O. Preparation of bio-nematicidal nanoparticles of *Eucalyptus officinalis* for the control of cyst nematode (*Heterodera sacchari*). Japs: Journal of Animal & Plant Sciences. 2020; 30(5).

[48] Sharmin S, Rahaman MM, Sarkar C, Atolani O, Islam MT, Adeyemi OS. Nanoparticles as antimicrobial and antiviral agents: A literature-based perspective study. Heliyon. 2021; 7(3): e06456. doi: 10.1016/j.heliyon.2021.e06456 33763612 PMC7973307

[49] Yien L, Zin NM, Sarwar A, Katas H. Antifungal activity of chitosan nanoparticles and correlation with their physical properties. International journal of Biomaterials. 2012. 10.1155/2012/632698 PMC3399401 22829829

[50] Gurunathan S, Han W., Kwon D.-N, Kim J.H. Enhanced antibacterial and anti-biofilm activities of silver nanoparticles against Gram-negative and Gram-positive bacteria. Nanoscale research letters. 2014; 9(1): 1–17. 25136281 10.1186/1556-276X-9-373 PMC4127560

[51] Gurunathan S, Han JW, Dayem AA, Eppakayala V, Kim JH. Oxidative stress-mediated antibacterial activity of graphene oxide and reduced graphene oxide in *Pseudomonas aeruginosa*. International journal of nanomedicine. 2012; 7: 5901. doi: 10.2147/IJN.S37397 23226696 PMC3514835

[52] Nagy A, Harrison A, Sabbani S, Munson RS Jr, Dutta PK, Waldman WJ. Silver nanoparticles embedded in zeolite membranes: release of silver ions and mechanism of antibacterial action. International journal of nanomedicine. 2011; 6: 1833. doi: 10.2147/IJN.S24019 21931480 PMC3173047

[53] Leung YH, Ng AM, Xu X, Shen Z, Gethings LA, Wong MT, et al. Mechanisms of antibacterial activity of MgO: non‐ROS mediated toxicity of MgO nanoparticles towards *Escherichia coli*. Small. 2014; 10(6): 1171–1183. doi: 10.1002/smll.201302434 24344000

[54] Gilbert P, McBain AJ. Potential impact of increased use of biocides in consumer products on prevalence of antibiotic resistance. Clinical microbiology reviews. 2003; 16(2): 189–208. doi: 10.1128/CMR.16.2.189-208.2003 12692093 PMC153147

[55] El Behiry A, Schlenker G, Szabo I, Roesler U. *In vitro* susceptibility of *Staphylococcus aureus* strains isolated from cows with subclinical mastitis to different antimicrobial agents. Journal of Veterinary Science. 2012; 13(2): 153–161. doi: 10.4142/jvs.2012.13.2.153 22705737 PMC3386340

[56] Xu Q, Hu X, Wang Y. Alternatives to Conventional Antibiotic Therapy: Potential Therapeutic Strategies of Combating Antimicrobial-Resistance and Biofilm-Related Infections. Molecular biotechnology. 2021; 63(12): 1103–1124. doi: 10.1007/s12033-021-00371-2 34309796

[57] Russell AD, Tattawasart U, Maillard JY, Furr J. Possible link between bacterial resistance and use of antibiotics and biocides. Antimicrobial agents and chemotherapy. 1998; 42(8): 2151–2151. doi: 10.1128/AAC.42.8.2151 9722471 PMC105894

[58] McDonnell G, Russell AD. Antiseptics and disinfectants: activity, action, and resistance. Clinical microbiology reviews. 1999; 12(1): 147–179. doi: 10.1128/CMR.12.1.147 9880479 PMC88911

[59] Kong B, Seog JH, Graham LM, Lee SB. Experimental considerations on the cytotoxicity of nanoparticles. Nanomedicine. 2011; 6(5): 929–941. doi: 10.2217/nnm.11.77 21793681 PMC3196306

[60] Sardari RRR, Zarchi SR, Talebi A, Nasri S, Imani S, Khoradmehr A, et al. Toxicological effects of silver nanoparticles in rats. African Journal of Microbiology Research. 2012; 6(27): 5587–5593.

[61] Gaillet S, Rouanet JM. Silver nanoparticles: their potential toxic effects after oral exposure and underlying mechanisms–a review. Food and Chemical Toxicology. 2015; 77: 58–63. doi: 10.1016/j.fct.2014.12.019 25556118

[62] Yong CQY, Valiyaveettil S, Tang BL. Toxicity of microplastics and nanoplastics in mammalian systems. International Journal of Environmental Research and Public Health. 2020; 17(5): 1509. doi: 10.3390/ijerph17051509 32111046 PMC7084551

[63] Hadrup N, Sharma AK, Loeschner K. Toxicity of silver ions, metallic silver, and silver nanoparticle materials after in vivo dermal and mucosal surface exposure: A review. Regulatory Toxicology and Pharmacology. 2018; 98: 257–267. doi: 10.1016/j.yrtph.2018.08.007 30125612

[64] Wen H, Dan M, Yang Y, Lyu J, Shao A, Cheng X, et al. Acute toxicity and genotoxicity of silver nanoparticle in rats. PloS one. 2017; 12(9): e0185554. doi: 10.1371/journal.pone.0185554 28953974 PMC5617228

[65] Khan MZ, Zahoor M. An overview of brucellosis in cattle and humans, and its serological and molecular diagnosis in control strategies. Tropical medicine and infectious disease. 2018; 3(2): 65. 10.3390/tropicalmed3020065 PMC6073575 30274461

[66] Alizadeh H, Salouti M, Shapouri R. Bactericidal effect of silver nanoparticles on intramacrophage brucella abortus 544. Jundishapur Journal of Microbiology. 2014; 7(3). doi: 10.5812/jjm.9039 25147682 PMC4138654

[67] Tamayo LA, Zapata PA, Vejar ND, Azócar MI, Gulppi MA, Zhou X, et al. Release of silver and copper nanoparticles from polyethylene nanocomposites and their penetration into *Listeria monocytogenes*. Materials Science and Engineering: C. 2014; 40: 24–31. doi: 10.1016/j.msec.2014.03.037 24857461

[68] Wu D, Fan W, Kishen A, Gutmann JL, Fan, B. Evaluation of the antibacterial efficacy of silver nanoparticles against *Enterococcus faecalis* biofilm. Journal of endodontics. 2014; 40(2): 285–290. doi: 10.1016/j.joen.2013.08.022 24461420

[69] Collins TL, Markus EA, Hassett DJ, Robinson JB. The effect of a cationic porphyrin on Pseudomonas aeruginosa biofilms. Current microbiology. 2010; 61(5): 411–416. doi: 10.1007/s00284-010-9629-y 20372908

[70] Lazar VJA. Quorum sensing in biofilms–how to destroy the bacterial citadels or their cohesion/power? Anaerobe. 2011; 17(6); 280–285. doi: 10.1016/j.anaerobe.2011.03.023 21497662

[71] Periasamy S, Joo HS, Duong AC, Bach THL, Tan VY, Chatterjee SS, et al. How *Staphylococcus aureus* biofilms develop their characteristic structure. Proceedings of the National Academy of Sciences. 2012; 109(4): 1281–1286. doi: 10.1073/pnas.1115006109 22232686 PMC3268330

[72] Rolim JP, De-Melo MA, Guedes SF, Albuquerque-Filho FB, De Souza JR, Nogueira NA, et al. The antimicrobial activity of photodynamic therapy against *Streptococcus mutans* using different photosensitizers. Journal of Photochemistry and Photobiology B: Biology. 2012; 106: 40–6. doi: 10.1016/j.jphotobiol.2011.10.001 22070899

[73] Rai M, Deshmukh SD, Ingle AP, Gupta IR, Galdiero M, Galdiero S. Metal nanoparticles: The protective nanoshield against virus infection. Critical reviews in microbiology. 2016; 42(1): 46–56. doi: 10.3109/1040841X.2013.879849 24754250

[74] Tian EK, Wang Y, Ren R, Zheng W, Liao W. Gold nanoparticle: recent progress on its antibacterial applications and mechanisms. Journal of Nanomaterials. 2021; 2021.

[75] Zhou W, Gao X, Liu D, Chen X. Gold nanoparticles for in vitro diagnostics. Chemical reviews. 2015; 115(19): 10575–636. doi: 10.1021/acs.chemrev.5b00100 26114396 PMC5226399

[76] Lee HE, Ahn HY, Mun J, Lee YY, Kim M, Cho NH, et al. Amino-acid-and peptide-directed synthesis of chiral plasmonic gold nanoparticles. Nature. 2018; 556(7701): 360–5. doi: 10.1038/s41586-018-0034-1 29670265

[77] Mba IE, Nweze EI. Nanoparticles as therapeutic options for treating multidrug-resistant bacteria: research progress, challenges, and prospects. World Journal of Microbiology and Biotechnology. 2021; 37(6): 1–30. 10.1007/s11274-021-03070-x PMC8159659 34046779

[78] Niño-Martínez N, Salas Orozco MF, Martínez-Castañó GA, Torres Méndez F, Ruiz F. Molecular mechanisms of bacterial resistance to metal and metal oxide nanoparticles. International journal of molecular sciences. 2019; 20(11): 2808. doi: 10.3390/ijms20112808 31181755 PMC6600416

[79] Hachicho N, Hoffmann P, Ahlert K, Heipieper HJ. Effect of silver nanoparticles and silver ions on growth and adaptive response mechanisms of *Pseudomonas putida* mt-2. FEMS microbiology letters. 2014; 355(1): 71–77. doi: 10.1111/1574-6968.12460 24801753

[80] Graves JL Jr, Tajkarimi M, Cunningham Q, Campbell A, Nonga H, Harrison SH, et al. Rapid evolution of silver nanoparticle resistance in *Escherichia coli*. Frontiers in genetics. 2015; 6: 42. doi: 10.3389/fgene.2015.00042 25741363 PMC4330922

[81] Huh AJ, Kwon YJ. Nanoantibiotics: a new paradigm for treating infectious diseases using nanomaterials in the antibiotics resistant era. Journal of Controlled Release. 2011; 156(2): 128–145. doi: 10.1016/j.jconrel.2011.07.002 21763369

[82] Karatzas KA, Webber MA, Jorgensen F, Woodward MJ, Piddock LJ, Humphrey TJ. Prolonged treatment of *Salmonella enterica serovar Typhimurium* with commercial disinfectants selects for multiple antibiotic resistance, increased efflux and reduced invasiveness. Journal of Antimicrobial Chemotherapy. 2007; 60(5): 947–955. doi: 10.1093/jac/dkm314 17855722

[83] Yang Y, Qin Z, Zeng W, Yang T, Cao Y, Mei C, et al. Toxicity assessment of nanoparticles in various systems and organs. Nanotechnology Reviews. 2017; 6(3): 279–289.

[84] Park EJ, Bae E, Yi J, Kim Y, Choi K, Lee SH, et al. Repeated-dose toxicity and inflammatory responses in mice by oral administration of silver nanoparticles. Environmental toxicology and pharmacology. 2010; 30(2): 162–8. doi: 10.1016/j.etap.2010.05.004 21787647

[85] Weldon BA M. Faustman E, Oberdörster G, Workman T, Griffith WC, Kneuer C, Yu IJ. Occupational exposure limit for silver nanoparticles: considerations on the derivation of a general health-based value. Nanotoxicology. 2016; 10(7): 945–56. doi: 10.3109/17435390.2016.1148793 26982810 PMC12091348

[86] Deen WM, Lazzara MJ, Myers BD. Structural determinants of glomerular permeability. American Journal of Physiology-Renal Physiology. 2001; 281(4): 579–596. doi: 10.1152/ajprenal.2001.281.4.F579 11553505

[87] Longmire M, Choyke PL, Kobayashi H. Clearance properties of nano-sized particles and molecules as imaging agents: considerations and caveats. 2008; 703–717. 10.2217/17435889.3.5.703 PMC3407669 18817471

