# Supplementary material for: Brucella species-induced brucellosis: Antimicrobial effects, potential resistance and toxicity of silver and gold nanosized particles
Source: PLoS One. 2022 Jul 14;17(7):e0269963. doi: 10.1371/journal.pone.0269963 (PMC9282596; doi:10.1371/journal.pone.0269963)
Supplement: S1 File — (PDF) [file pone.0269963.s001.pdf]

SPRINGER NATURE

Author Services

Editing Certificate

This document certifies that the manuscript

Brucella species-induced Infectious Brucellosis: Antimicrobial effects, Potential resistance and Toxicity of Silver and Gold Nano-sized Particles

prepared by the authors

Ayman Elbehiry

was edited for proper English language, grammar, punctuation, spelling, and overall style by one or more of the highly qualified native English speaking editors at SNAS.

This certificate was issued on **May 25, 2022** and may be verified on the SNAS website using the verification code **A37A-67AA-426F-D49D-A5F0**.

Neither the research content nor the authors' intentions were altered in any way during the editing process. Documents receiving this certification should be English-ready for publication; however, the author has the ability to accept or reject our suggestions and changes. To verify the final SNAS edited version, please visit our verification page at secure.authorservices.springernature.com/certificate/verify.
If you have any questions or concerns about this edited document, please contact SNAS at support@as.springernature.com.

SNAS provides a range of editing, translation, and manuscript services for researchers and publishers around the world.
For more information about our company, services, and partner discounts, please visit authorservices.springernature.com.
